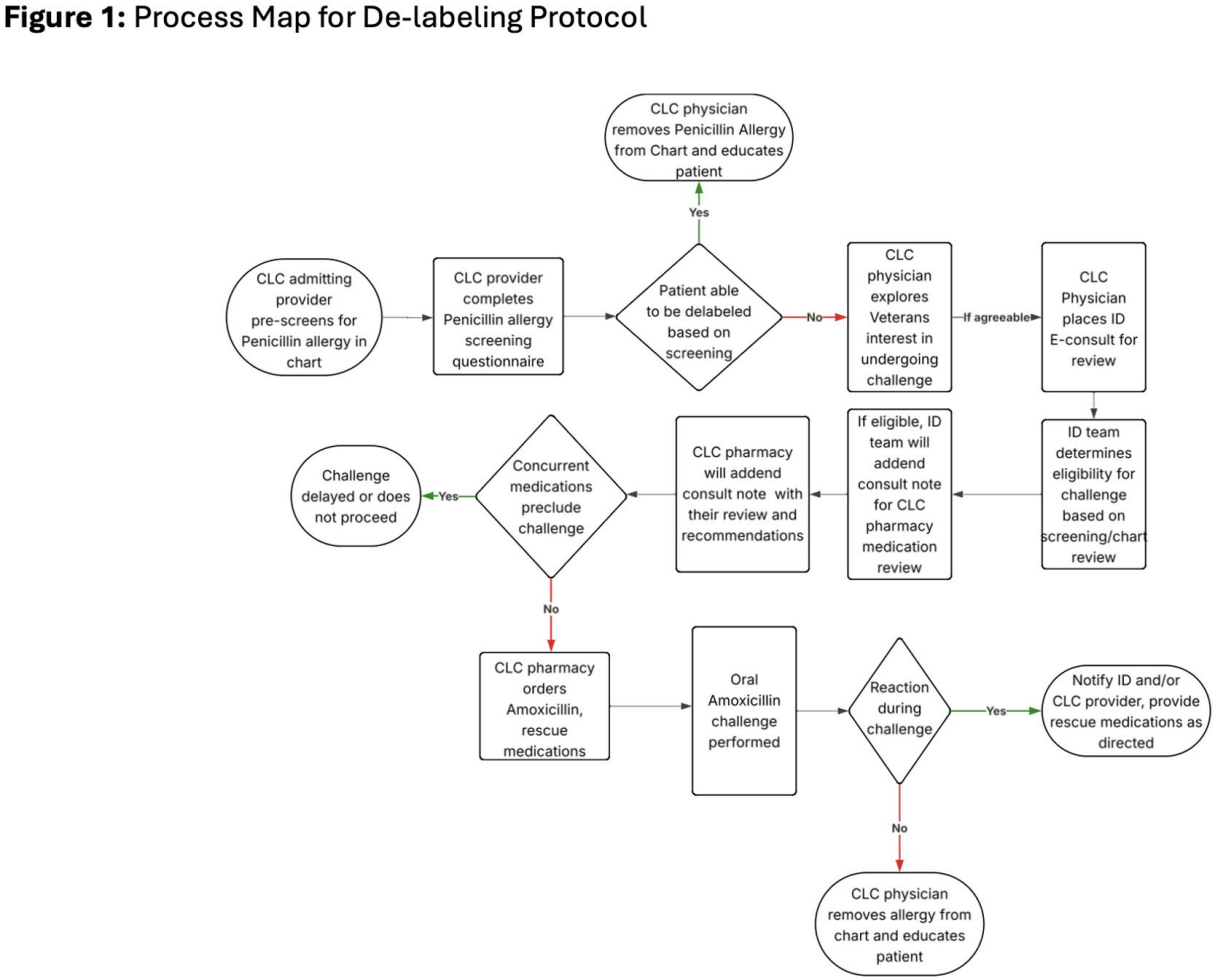# 88 Impact of a Multi-pronged Antimicrobial Stewardship Intervention on Antibiotic Prescribing for Pediatric Acute Bacterial Rhinosinusitis

**DOI:** 10.1017/ash.2026.10513

**Published:** 2026-06-23

**Authors:** Ellen Earle, Kimberly Mackay, Monica K Sikka, Christopher Pfeiffer, David Zhang, Jason Roberts, Margaret Smithpeter, Kristina L. Bajema

**Affiliations:** 1 Oregon Health and Science University; 2 Portland VA Medical Center; 3 VA Portland Health Care System; 4 Portland VA Community Living Center

## Abstract

**Background:** Penicillin allergies are common, reported in roughly 10% of the U.S. population, but fewer than 1% have a true IgE-mediated allergy. Penicillin allergy labels are associated with increased use of broad-spectrum antibiotics, higher healthcare costs, and the development of antimicrobial resistance. Antibiotic stewardship efforts have focused on identifying and de-labeling individuals without a true penicillin allergy. To date, most penicillin allergy de-labeling initiatives have been implemented in acute-care settings, where time constraints and clinical acuity may limit feasibility. Long-term care facilities represent a unique opportunity for penicillin allergy de-labeling, as residents often have prolonged stays and are less acutely ill. Previously, we found that 14% of residents admitted to the Veterans Affairs (VA) Community Living Center (CLC) in Vancouver, Washington had a documented allergy to a penicillin-class antibiotic. Here, we describe design and implementation of a penicillin allergy de-labeling program at a VA CLC and highlight opportunities for multidisciplinary collaboration. **Methods:** This antibiotic stewardship initiative was developed through collaboration between the VA Portland antimicrobial stewardship team and CLC providers, including physicians, nursing staff, and pharmacy. A standardized protocol was created to identify residents eligible for penicillin allergy evaluation and to outline steps for a direct oral challenge (Figure 1). At admission, CLC providers are instructed to identify residents with electronic health record (EHR) documentation of a penicillin class allergy and complete a structured allergy screening questionnaire. Residents with antibiotic intolerance alone (e.g., nausea and vomiting) receive education, and the penicillin allergy is removed from the EHR. Residents who require and agree to further evaluation are referred via electronic consult to the infectious diseases team, who verifies eligibility to undergo a direct oral challenge. CLC pharmacists review resident medications for any contraindications to performing a challenge, including receipt of medications that would reduce the histamine response. Eligible residents undergo a direct oral amoxicillin challenge with a single 500-mg dose and are monitored by nursing staff for 60 minutes. Patients without evidence of reaction receive education, and the penicillin allergy is removed from the EHR. Planned Evaluation: Planned outcome measures include completion of allergy screening, de-labeling based on chart review alone, eligibility for and completion of oral challenge, challenge outcome, and impact on subsequent antibiotic prescribing. Conclusion:This initiative describes a structured, reproducible framework for implementing penicillin allergy de-labeling in a VA post-acute care setting. The program has the potential to improve antibiotic selection and strengthen antimicrobial stewardship in skilled nursing facility (SNF) enviornments.

**Figure:**